# Prevalence and Severity of Menopausal Symptoms and the Quality of Life in Middle-aged Women: A Study from Sri Lanka

**DOI:** 10.1155/2019/2081507

**Published:** 2019-07-01

**Authors:** Nirmala Rathnayake, Janaka Lenora, Gayani Alwis, Sarath Lekamwasam

**Affiliations:** ^1^Department of Nursing, Faculty of Allied Health Sciences, University of Ruhuna, Sri Lanka; ^2^Department of Physiology, Faculty of Medicine, University of Ruhuna, Sri Lanka; ^3^Department of Anatomy, Faculty of Medicine, University of Ruhuna, Sri Lanka; ^4^Population Health Research Centre, Department of Medicine, Faculty of Medicine, University of Ruhuna, Sri Lanka

## Abstract

Menopausal symptoms and quality of life (QOL) of pre- and postmenopausal women in Sri Lanka have not been studied adequately. This study aimed to evaluate the prevalence and severity of menopausal symptoms and the QOL of pre- and postmenopausal women in Galle District, Sri Lanka. A cross-sectional study was conducted with a randomly selected sample of premenopausal (n=184) and postmenopausal (n=166) community-dwelling healthy women aged 30-60 years. The mean (SD) ages of pre- and postmenopausal women, respectively, were 46.1(3.7) and 55.8(3.8) years. Menopausal symptoms were evaluated using the menopause rating scale under three subscales: psychological symptoms, somatovegetative symptoms, and urogenital symptoms. The QOL was evaluated using the short form 36 survey under eight domains. Further, sociodemographic status, gynaecologic factors, physical activity pattern (walking, moderate, and vigorous), body mass index, and waist to hip ratio were also evaluated. The prevalence and severity of all the menopausal symptoms were higher among postmenopausal women. In premenopausal women, the most frequently reported menopausal symptoms were mental exhaustion (49.5%), joint and muscular discomforts (48.5%), and irritability (41.3%). Physical and mental exhaustion (53%), irritability (48.2%), depressive mood (43.4%), and hot flushes (42.2%) were the most frequently reported menopausal symptoms in postmenopausal women. The QOL was significantly impaired among postmenopausal women [mean (SD); 57.47(18.83)] compared to premenopausal women [mean (SD); 66.82(17.93)] (p<0.001). Psychological symptoms score and somatovegetative symptoms score were associated with the QOL of premenopausal women (adjusted R^2^; 0.35). Somatovegetative symptoms score, psychological symptoms score, moderate and vigorous physical activity scores, and monthly income were associated with the QOL in postmenopausal women (adjusted R^2^; 0.38). The current study showed that the prevalence and severity of menopausal symptoms and impaired QOL were significantly higher among postmenopausal women, compared to premenopausal women. Menopausal symptoms mostly contributed to the poorer QOL in both pre- and postmenopausal women.

## 1. Introduction 

Menopause is a natural process that every woman experiences due to the age-related gradual decline of primordial ovarian follicles. It is the permanent cessation of menstruation and is defined as 12-month amenorrhea after the final menstruation [1] with no other attributable cause. Menopause and associated biological changes have a negative impact on the general health and quality of life (QOL) as well as the wellbeing of middle-aged women [2–4].

Menopausal symptoms and their severity vary from person to person due to the effects of confounding factors [5] such as lifestyle, social status, body composition, and psychological status [6]. Menopausal symptoms, especially the vasomotor and sexual symptoms, are associated with impaired QOL in women [2, 7]. QOL is “an individual's perception of their position in life in the context of the culture and value systems in which they live and in relation to their goals, expectations, standards and concerns” [8]. It is an imperative outcome measure of overall health. Therefore, understanding the impact of menopause on the QOL in middle-aged women is critically important in the contemporary health care system [9].

The life expectancy of women is increasing worldwide due to the scientific and technological advances. The average age of a Sri Lankan woman attaining menopause is between 49 and 51 years [2, 10]. Considering the female life expectancy of 78 years, a woman has to spend approximately three decades of her life in the postmenopausal period. Therefore, overall health and wellbeing of middle-aged women have become a major global public health concern. However, there is a paucity of studies [2, 10] related to the menopausal symptoms and the QOL in pre- and postmenopausal women in Sri Lanka. Therefore, this study was designed to evaluate the prevalence and severity of menopausal symptoms, the QOL, and the factors that determine the QOL in a group of pre- and postmenopausal women selected from Galle District, Sri Lanka.

## 2. Materials and Methods 

### 2.1. Study Design, Subjects, and Setting

This descriptive cross-sectional study included 184 premenopausal and 166 postmenopausal community-dwelling healthy women aged 30-60 years, selected from the Bope-Poddala Medical Officer of Health (MOH) area, Galle District in Southern Province of Sri Lanka. The study was conducted from June 2015 to January 2017 as a part of a study project titled “Effects of menopause on bodily structure, functions and physical health” carried out at the Faculty of Medicine, University of Ruhuna, Sri Lanka. The sample size for the main study was calculated based on the BMI (means and SD) between pre- and postmenopausal women in a previous Chinese study [11], using the formula N = (Z_*α*/2_+Z_*β*_)^2^*∗* (*σ*1^2^ + *σ*2^2^)/ (*μ*1 – *μ*2)^2^, where Z_*α*/2_= 95% confidence interval = 1.96, Z_*β*_= 80% power = 0.84, *σ*1, *σ*2 = standard deviations, *μ*1 – *μ*2 = effects size/ difference between two means. Minimum recommended number of premenopausal and postmenopausal women in each category was 155.

The cluster sampling method was used to achieve the required study sample from 5 out of 18 public health midwives' (PHM) areas that are under the administration of Bope-Poddala MOH office. Cluster sizes for pre- and postmenopausal women were considered based on the total number of pre- and postmenopausal women resident in the specific areas. Support of the Public Health Midwife (primary health care provider) and the Grama Niladari (primary administrative officer) of the specific areas were obtained to select the sample.

Women who were pregnant or lactating or suffering from noncommunicable diseases (NCD), acute or chronic surgical conditions, and polycystic ovarian syndrome (PCOS) were excluded. Women who could not understand the questionnaire, refused to participate in the study, and were illiterate as well as women on hormone replacement therapy (HRT) or hormonal contraceptives were excluded from the study. Menopausal status was considered on the self-stated menstrual history based on the classification of Stages of Reproductive Aging Workshop (STRAW) [12]. Women with the cessation of menstruation within the previous 12 months after last menstruation were considered as postmenopausal women.

### 2.2. Data Collection

A background questionnaire that was self-developed and pretested was used to evaluate the sociodemographic, gynaecologic, and obstetric characteristics. The presence and severity of menopausal symptoms were evaluated using the Menopause Rating Scale (MRS) [13, 14], a culturally adopted version used in a previous Sri Lankan study [2]. It includes eleven symptoms under three subscales of symptoms, namely, psychological symptoms (disturbances of women's psychological status such as depressive mood, irritability, anxiety, and physical and mental exhaustion), somatovegetative symptoms (disturbances of women's physical and functional status such as hot flushes/sweating, heart discomfort, sleep problems, and joint and muscular discomforts), and urogenital symptoms (disturbances of women's urinary and sexual status such as sexual problems, bladder problems, and dryness of vagina). These were evaluated in a five-point severity scale by way of none, mild, moderate, severe, and very severe. Overall MRS score was generated by summing up the scores given for eleven symptoms. The subscales' scores were also generated by summing up the relevant scores for each symptom that were given in five-point Likert scale as none = 0, mild = 1, moderate = 2, severe = 3, and very severe = 4 [15].

The QOL was evaluated using the Short Form (SF) 36 survey [16], a validated tool for Sri Lankan context [17]. It includes eight domains of QOL, namely, physical functioning (limitations of day to day activities in a typical day), role performance due to physical health (problems associated with work or other regular daily activities as a result of physical health), role performance due to emotional problems (problems associated with work or other regular daily activities as a result of any emotional problems such as feeling depressed or anxious), social functioning (extent of physical health or emotional problems that interfered with normal social activities with family, friends, neighbours, or groups), emotional wellbeing (feelings and reactions), comfort/perception of pain (extent of bodily pain felt and its interference on physical and psychological status), vitality/perception of energy or fatigue (extent of interference with routine physical and emotional problems), and general health (feelings regarding the general health). Further, the physical and psychological dimension scores are formulated by summing up the respective domain scores [16]. In this questionnaire, each domain was given a score ranging from 0 to 100 using the original coding algorithm [16]. The higher scores indicate the higher level of QOL in each domain and overall QOL.

The physical activity level was estimated with the International Physical Activity Questionnaire (IPAQ), short version which was forward-backward translated into the Sinhala language and pretested. Participants were asked to report the time they were involved in walking, moderate intensity activity, and vigorous intensity activity during the last week prior to the interview. The physical activity data were converted to minutes per week and expressed as a metabolic equivalent (MET-min/week) according to the IPAQ guidelines for data processing [18].

MRS and SF 36 survey questionnaires were self-administered and background questionnaire and IPAQ were interviewer-administered in nature. Women who were unable to provide accurate answers on their own, especially those who had poor understanding and visual impairments, were supported by the principal investigator without suggesting what to include or directing them to come up with answers. Each questionnaire was rechecked by the principal investigator for any missing data in order to ensure its completeness before they left.

Body weight was measured while fasting, with empty bladder, to the nearest 0.1 kg with women dressed in light cloths while height was determined without shoes to the nearest 0.1 cm using a stadiometer with a beam balance (NAGATA, Tainan, Taiwan). Circumferences (cm) of waist (WC) and hip (HC) were measured with a plastic nonstretchable tape. All anthropometry indices were obtained adhering to standard protocols [19] by the principal investigator to ensure the consistency of each measurement. Circumferences were read three times with 1 mm measurement consistency among each measurement and an average of three measurements was obtained. Body mass index (BMI, kg/m^2^) and waist to hip ratio (WHR) were calculated.

### 2.3. Statistical Analyses

Continuous variables were presented as mean (SD) and categorical variables were presented as frequencies (%). Chi square test of independence was performed to analyze the association of menopausal status with prevalence and severity of menopausal symptoms. Presence or absence of menopausal symptoms was separately calculated and the women who presented with menopausal symptoms were further categorized into two severity groups as mild to moderate and severe to very severe. Menopausal symptoms scores and the QOL scores were calculated and independent sample t-test was applied to detect the differences between pre- and postmenopausal women.

Independent sample t-test and one way ANOVA with Tukey's test were used to determine the associations between the QOL and categorical (sociodemographic and gynecologic or obstetric) variables. Pearson (r) or Spearman (rho) or point biserial correlation coefficient verified the associations or correlations between the overall QOL score and evaluated variables in both pre- and postmenopausal women. The variables which showed significant correlations with the QOL were further analyzed by multiple regression analysis to ensure the factors affecting the QOL. Hierarchical multiple regression was performed again while controlling the effect of confounders for the QOL: age in both groups and age at menopause and time since menopause in postmenopausal women.

SPSS 20.0 version was used in the data analyses process and P value <0.05 was considered as statistically significant.

### 2.4. Ethical Clearance

The Ethics Review Committee, Faculty of Medicine, University of Ruhuna, Sri Lanka, granted the ethical clearance for this study (reference number: 24.09.2014:3.2). Each participant signed the written informed consent before answering the questionnaire.

## 3. Results

### 3.1. Basic Characteristics of Pre- and Postmenopausal Women

The mean (SD) ages of pre- and postmenopausal women were 46.1(3.7) and 55.8(3.8) years, respectively (p<0.001). The majority of women were Sinhalese, married, and living with their families in both pre- and postmenopausal groups (Table 1). The mean (SD) ages of menopause and time since menopause of postmenopausal women were 48.3(3.9) and 7.4(5.0) years, respectively. There was no statistically significant difference between the two groups except the height (postmenopausal women were shorter than premenopausal women) (Table 1).

### 3.2. Prevalence and Severity of Menopausal Symptoms in Pre- and Postmenopausal Women

The prevalence of at least one menopausal symptom among pre- and postmenopausal women was 90.8% (167) and 96.4% (160), respectively (p<0.001). Prevalence and severity of symptoms were higher among postmenopausal women (Table 2). The frequently reported menopausal symptoms among premenopausal women were physical and mental exhaustion (49.5%), joint and muscular discomforts (48.5%), and irritability (41.3%) of mild to moderate severity. In postmenopausal women, physical and mental exhaustion (53%), irritability (48.2%), depressive mood (43.4%), and hot flushes (42.2%) of mild to moderate severity were observed. Severe symptoms were more prevalent among postmenopausal women compared to premenopausal women. Further, 47.6% of postmenopausal women reported joints and muscular discomforts of severe to very severe intensity. Presence of hot flushes (p<0.001), sleep disturbances (p<0.001), anxiety (p=0.03), physical and mental exhaustion (p<0.001), sexual problems (p=0.03), bladder problems (p<0.001), dryness of vagina (p<0.001), and joint and muscular discomforts (p<0.001) were more frequent among postmenopausal women compared to premenopausal women (Table 2).

The mean (SD) overall and subscales of symptoms scores were higher among postmenopausal women compared to premenopausal women (p<0.001) (Table 3).

### 3.3. Quality of Life and Associated Factors of Pre- and Postmenopausal Women

The mean (SD) overall QOL and domains of QOL scores were lower among postmenopausal women (p<0.001). Significantly lower QOL was observed in few domains, namely, physical functioning (p<0.001), role performance due to physical health (p<0.001), role performance due to emotional problems (p=0.005), and comfort (perception of pain) (p=0.001) domains in postmenopausal women (Table 3).

**Figure 1:**
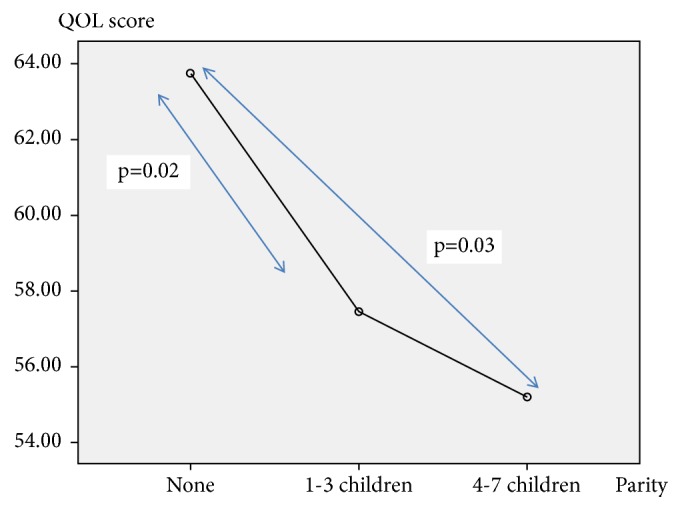
Mean plot for the association between parity and QOL in postmenopausal women (one way ANOVA tukey's test).

When the associations between QOL and sociodemographic and gynecologic variables were evaluated, there were no significant associations found in premenopausal women. Only monthly income and parity showed significant associations with the QOL in postmenopausal women (Table 4). High monthly income associated with higher QOL while high parity (>4 children) (Figure 1) associated with lower QOL in postmenopausal women (monthly income: rho; 0.24, p=0.006 and parity: rho; -0.21, p=0.03) (Table 5).

All the individual scores of menopausal symptoms except dryness of vagina and symptom subscale scores showed negative correlations with the QOL in both pre- and postmenopausal women. Moderate, vigorous, and total physical activity scores showed positive correlations with the QOL of postmenopausal women (Table 5). Adjusting the above associations for current age in both groups and age at menopause and time since menopause in postmenopausal women did not change the strength of associations materially.

In multiple regression analysis, psychological symptoms score and somatovegetative symptoms score remained as main two factors associated with the QOL of premenopausal women accounting for 35% of variance (adjusted R^2^=0.35). Among the postmenopausal women, somatovegetative symptoms score, psychological symptoms score, moderate and vigorous physical activity scores, and monthly income showed significant associations with the QOL accounting for 38% of variance (adjusted R^2^=0.38). Psychological symptoms score was the strongest factor associated with the QOL in premenopausal women (R:-0.56, R^2^=0.31) while somatovegetative symptoms score emerged as the strongest factor in postmenopausal women (R:-0.49, R^2^=0.24). The above variances remained unchanged even after controlling for possible confounders (age in all and age at menopause and time since menopause in postmenopausal women) in hierarchical multiple regression.

## 4. Discussion 

This community-based cross-sectional survey revealed a high prevalence and severity of menopausal symptoms in postmenopausal women leading to impairment of QOL compared to premenopausal women in Galle District, Sri Lanka. The most frequently annoying menopausal symptoms among pre- and postmenopausal women were psychological and somatovegetative in nature. Joint and muscular discomforts with “severe” to “very severe” intensity and depressive mood in “mild” to “moderate” severity were more prevalent among postmenopausal women. Hot flushes and urogenital complaints, however, were infrequent among them. This led to impairment of overall QOL in postmenopausal women. In postmenopausal women, the QOL was associated with menopausal symptoms, physical activity, and monthly income. In premenopausal women, only the menopausal symptoms were associated with the QOL.

Similar findings of prevalence of menopausal symptoms have been reported in previous studies carried out in Sri Lanka [2, 10] as well as in other different communities [20, 21] including many Asian countries [3, 7, 22–24]. However, studies from the Western countries have reported [4, 9, 20, 25–27] higher occurrence of hot flushes, night sweats, decreased interest in sex, and urogenital problems that were less common in our study.

Several studies [2, 26, 28] have reported that impairment of QOL can be mainly limited to few domains. The women in current study, however, reported impaired QOL in several domains. The negative correlations between menopausal symptoms scores and overall QOL score have been seen earlier [2, 5, 7, 29, 30]. Marital status, educational level, and social and economic level have been observed to positively affect QOL while advanced age and the number of children who live with the family have been observed as negatively affecting factors on the QOL of postmenopausal women [5, 7, 31, 32].

The prevalence of menopausal symptoms may show a geographical variation. In tropical countries such as Sri Lanka, women may not distinguish hot flushes from hot weather spells. Further, the sensitivity of women to menopausal symptoms may be affected by ethnic backgrounds, religious beliefs, geographical variations, cultural variations, and lifestyle factors [28]. Further, as urogenital and sexual issues are not discussed openly in the Asian culture, they might get translated to somatic and psychological symptoms. Also, they might believe those symptoms are a part of ageing.

The impairment of QOL in multiple domains which has been observed could partly be explained with the usage of self-administered survey tools. Participants may have answered many items to express their feelings. Previous studies in Sri Lanka [2, 10] have used interviewer based discussions for data collection and probably did not allow overlap of responses.

Positive association of higher monthly income and negative association of higher number of children in family with the QOL in postmenopausal women are explainable. Higher monthly income would provide better economic background, coping abilities, and positive perceptions and in turn better QOL. More children in the family would add more worries and responsibilities resulting in poor QOL [32]. Further, high level of physical activity is likely to enhance the QOL as physical activity is associated with positive attributes in life. Physical activity has been shown to be a predictor of QOL [33] of postmenopausal women as the postmenopausal women gained weight during this period which again has a negative impact on the QOL [21].

Central and intra-abdominal fat accumulation is relatively higher among postmenopausal women. Higher BMI has shown a negative impact on physical domain of QOL among postmenopausal women [34] while better QOL was seen among thin women [35]. However, in this study, BMI and WHR were not associated with QOL of postmenopausal women.

This study provided valuable information on the enhancement of QOL of middle-aged women in Sri Lanka. We identified that the QOL is mainly impaired by menopausal symptoms such as psychological symptoms, namely, irritability, physical and mental exhaustion, etc. The impact of symptoms that are directly related to the estrogen depletion such as hot flushes (vasomotor symptoms) and urogenital symptoms on the QOL is less. Further, lifestyle factors such as physical activity are vital to enhance the QOL. Therefore, the interventions focused on enhancement of the QOL of middle-aged women should be targeted towards lifestyle changes and behavioral modifications. These lifestyle changes should also include psychological adjustments to menopause and strategies to cope with menopause rather than treating women with HRT or other medications.

We identified several strengths and limitations of this study. We evaluated women aged 30-60 years who were apparently healthy. This approach minimizes the confounding effects of ageing and comorbidities towards the QOL. Further, generalizability of the findings is limited to other areas of the country due to the geographical variations in socioeconomic status among women in Sri Lanka.

## 5. Conclusions 

This study revealed that prevalence of menopausal symptoms and their severity were significantly higher among postmenopausal women compared to premenopausal women. The overall QOL and scores of some domains, namely, physical functioning, role performance due to emotional and physical problems, and comfort (perception of pain), were significantly impaired in postmenopausal women compared to premenopausal women. The psychological symptoms score and somatovegetative symptoms score were associated with the QOL of premenopausal women. In postmenopausal women, somatovegetative symptoms score, psychological symptoms score, moderate and vigorous physical activity scores, and monthly income were significantly associated with the QOL.

## Figures and Tables

**Table 1:** Characteristics of pre and postmenopausal women (n=350).

Characteristics	Sub category	Premenopausal women (n=184) Mean (SD) or Frequency (%)	Postmenopausal women (n=166) Mean (SD) or Frequency (%)	P value
*Sociodemographic characteristics*				

Age (years)		46.1(3.74)	55.8(3.80)	<0.001*∗*

Ethnicity	Sinhala	171 (92.9)	160(96.4)	0.11
	Non Sinhala	13 (7.1)	6(3.6)	

Employment status	Employed	58(31.5)	49(29.3)	0.38
	Non employed	126(68.5)	118(70.7)	

Civil status	Married	169(91.8)	125(74.9)	<0.001
	Single or widowed or divorced	15(8.2)	42(25.1)	

Living companion	With husband and children	134(72.8)	103(61.6)	0.003
	With Husband or Children	16(8.6)	35(21.0)	
	Alone or Others	34(18.5)	29(17.4)	

Education status	Primary education	37(20.1)	46(27.6)	0.12
	Secondary education	68(37.0)	64(38.3)	
	Upper secondary education, degree or diploma	79(42.9)	57(34.1)	

Monthly income	Below 20000 LKR	92(50.0)	125(74.8)	<0.001
	Above 20000 LKR	92(50.0)	42(25.2)	

*Gynecologic factors*				

Age at menopause (years)		-	48.3(3.98)	-

Time since menopause (years)		-	7.4(5.04)	-

Parity	Nulliparous	12(6.5)	23(13.9)	0.02
	1-3 children	152(82.6)	97(58.4)	
	4-7 children	20(10.9)	46(27.7)	

Modes of deliveries	None	12(6.5)	23(13.9)	0.006
	NVD	109(59.2)	110(66.3)	
	LSCS	40(21.7)	23(13.9)	
	NVD and LSCS	23(12.5)	10(6.0)	

*Other evaluated variables*				

Weight (kg)		58.0(9.8)	57.0(11.9)	0.44*∗*

Height (m)		1.5(0.1)	1.49(0.1)	<0.001*∗*

WC (cm)		82.5(9.8)	83.3(12.3)	0.50*∗*

HC (cm)		97.0(8.5)	98.7(10.1)	0.09*∗*

WHR		0.84(0.1)	0.84(0.1)	0.47*∗*

BMI (kg/m^2^)		24.98(4.02)	25.98(4.56)	0.37*∗*

Walking score (MET/min/week)		829.21(186.74)	580.28(156.89)	0.01*∗*

Moderate physical activities score (MET/min/week)		4868.04(574.20)	4770.12(857.01)	0.20*∗*

Vigorous physical activities score (MET/min/week)		1785.21(1784.93)	2297.59(1917.41)	0.01*∗*

Total physical activity score (MET/min/week)		7482.51(2400.03)	7648.03(2534.65)	0.53*∗*

SD=standard deviation, NVD=normal vaginal deliveries, LSCS=lower segment caesarian section, BMI=body mass index, WC=waist circumference, HC=hip circumference, WHR=waist to hip ratio

150 LKR = 1 USD (LKR=Sri Lankan rupees)

Primary education= grade 1-10, secondary education=GCE ordinary level

Differences between two groups were compared with independent sample t test*∗* and chi square test.

**Table 2:** Prevalence and severity of menopausal symptoms in pre and postmenopausal women (n=350).

Menopausal symptom	Premenopausal women (n=184)			Postmenopausal women (n=166)			P value
	None n(%)	M-M n(%)	S-VS n(%)	None n(%)	M-M n(%)	S-VS n(%)	
Hot flushes, sweating	113 (61.4)	65 (35.3)	6 (3.3)	75 (45.2)	70 (42.2)	21 (12.7)	<0.001

Heart discomfort	132 (71.7)	47 (25.5)	5 (2.7)	104 (62.1)	53 (15.9)	9 (5.4)	0.14

Sleep problems	129 (70.1)	46 (25)	9 (4.9)	69 (41.6)	65 (39.2)	32 (19.3)	<0.001

Depressive mood	104 (56.5)	69 (37.5)	11 (6.0)	80 (48.2)	72 (43.4)	14 (8.4)	0.26

Irritability	101 (54.9)	76 (41.3)	7 (3.8)	77 (46.4)	80 (48.2)	9 (5.4)	0.26

Anxiety	114 (62)	61 (33.2)	9 (4.9)	80 (48.2)	74 (44.6)	12 (7.2)	0.03

Physical and mental exhaustion	82 (44.6)	91 (49.5)	11 (6.0)	45 (27.1)	88 (53.0)	33 (19.9)	<0.001

Sexual problems	149 (81.0)	31 (16.8)	4 (2.2)	115 (32.9)	43 (25.9)	8 (4.8)	0.03

Bladder problems	151 (82.1)	27 (14.7)	6 (3.3)	97 (58.4)	56 (33.7)	13 (7.8)	<0.001

Dryness of vagina	144 (78.3)	36 (19.6)	4 (2.2)	111 (66.9)	45 (27.1)	10 (6.0)	<0.001

Joint and muscular discomfort	40 (21.7)	89 (48.4)	55 (29.9)	20 (12.0)	67 (40.4)	79 (47.6)	<0.001

M-M = mild to moderate, S-VS = severe to very severe

Associations were compared with chi square test of independence.

**Table 3:** Menopausal symptoms scores and QOL scores of pre and postmenopausal women (n=350).

Characteristics	Premenopausal women (n=184) Mean (SD)	Postmenopausal women (n=166) Mean (SD)	P value
*Menopausal symptoms scores*			

Psychological symptoms score	2.78(3.10)	4.03(3.22)	<0.001

Somato-vegetative symptoms	3.14(2.68)	5.16(3.01	<0.001

Uro-genital symptoms	0.96(1.72)	1.77(2.21)	<0.001

Overall MRS score	6.90(6.20)	10.98(6.90)	<0.001

*Quality of life scores*			

Physical functioning	81.68(20.49)	65.35(24.46)	<0.001

Role performance due to physical health	62.09(42.14)	36.60(42.96)	<0.001

Role performance due to emotional problems	61.24(43.59)	47.61(45.86)	0.005

Vitality (perception of energy/fatigue)	62.23(18.44)	58.43(20.41)	0.06

Emotional wellbeing	71.57(17.71)	70.99(18.53)	0.76

Social function	73.17(23.68)	68.37(24.17)	0.06

Comfort (perception of pain)	66.68(23.11)	58.50(23.62)	0.001

General health	55.95(17.18)	53.92(17.43)	0.27

Physical health dimension	65.48(18.31)	53.57(19.45)	<0.001

Psychological health dimension	68.16(19.78)	61.36(21.16)	0.002

Overall QOL	66.82(17.93)	57.47(18.83)	<0.001

MRS=menopause rating scale, QOL=quality of life

Differences between two groups were compared with independent sample t test.

Higher scores indicate higher level of QOL in each domain and overall QOL.

**Table 4:** Association between QOL and evaluated categorical (sociodemographic and gynecologic) variables of pre- and postmenopausal women (n=350).

Variable	Premenopausal women (n=184)			Postmenopausal women (n=166)	
	Subcategory	QOL Mean (SD)	P value	QOL Mean (SD)	P value
*Sociodemographic status*					

Ethnicity	Sinhala	66.81(17.80)	0.98*∗*	57.41(18.37)	0.91*∗*
	Non-Sinhala	66.95(20.37)		58.90(31.08)	

Educational status	Primary education	64.32(19.69)	0.20*∗∗*	53.79(20.84)	0.21*∗∗*
	Secondary education	65.06(19.53)		57.60(17.83)	
	Upper secondary education, degree, or diploma	69.51(15.34)		60.33(18.02)	

Employment status	Nonemployed	67.07(18.29)	0.78*∗*	57.40(18.54)	0.94*∗*
	Employed	66.28(17.27)		57.63(19.70)	

Civil status	Married	67.34(17.90)	0.18*∗*	58.59(18.50)	0.18*∗*
	Others	60.96(17.82)		54.13(19.64)	

Monthly income	< 20000.00LKR	64.76(19.15)	0.12*∗*	55.17(18.64)	0.007*∗*
	> 20000.00LKR	68.88(16.48)		64.23(17.95)	

Living companion	Husband and Children	66.13(17.35)	0.16*∗∗*	58.13(18.19)	0.81*∗∗*
	Husband or Children	62.32(23.25)		55.81(18.04)	
	Alone or Others	71.65(16.99)		57.22(22.28)	

*Gynecological and reproductive factors*					

Parity	None	76.71(15.68)	0.05*∗∗*	60.83(19.88)	0.04*∗∗*#
	1-3 children	66.84(17.60)		59.43(17.75)	
	4-7 children	60.78(19.81)		51.65(19.66)	

Mode of delivery	None	76.71(15.68)	0.23*∗∗*	60.83(19.88)	0.39*∗∗*
	NVD	66.56(17.65)		55.80(18.74)	
	LSCS	66.17(18.05)		62.07(16.78)	
	Both NVD and LSCS	64.05(19.58)		57.51(21.83)	

QOL: health related quality of life; NVD: normal vaginal delivery; LSCS: lower segment cesarean section.

*∗* QOL among the groups was compared with independent sample t-test in the variables with two categories.

*∗∗* QOL among the groups was compared with one-way ANOVA test in the variables with three or more categories.

#Post hoc analysis with Tukey's test; the difference was observed between the following groups.

None – 4-7 children; p=0.03

1-3 children – 4-7 children; p=0.02

**Table 5:** Correlation between QOL and evaluated variables of pre- and postmenopausal women (n=350).

Variable	Premenopausal women (n=184)	Postmenopausal women (n=166)
	Correlation coefficient	Correlation coefficient
Ethnicity ^a^	-0.004 (ns)	0.01 (ns)

Educational status^b^	0.11 (ns)	0.14 (ns)

Employment status^a^	-0.02 (ns)	0.14 (ns)

Civil status^a^	-0.10 (ns)	-0.08 (ns)

Monthly income^a^	0.10 (ns)	0.24 *∗∗*

Living companion^b^	0.13 (ns)	0.03 (ns)

Parity^b^	0.08 (ns)	-0.21 *∗*

Mode of delivery^b^	-0.10 (ns)	0.01 (ns)

Psychological symptoms score ^c^	-0.56*∗∗∗*	-0.47*∗∗∗*

Somatovegetative symptoms score ^c^	-0.50*∗∗∗*	-0.49*∗∗∗*

Urogenital symptoms score ^c^	-0.22*∗∗*	-0.27*∗∗∗*

Overall MRS score ^c^	-0.56*∗∗∗*	-0.52*∗∗∗*

Age (years)^c^	-0.04 (ns)	-0.09 (ns)

BMI (kg/m^2^)^c^	-0.06 (ns)	-0.05 (ns)

WHR ^c^	0.02 (ns)	-0.08 (ns)

Walking score (MET/min/week)^c^	0.03 (ns)	0.14 (ns)

Moderate physical activities score (MET/min/week)^c^	0.14 (ns)	0.26*∗∗*

Vigorous physical activities score (MET/min/week)^c^	0.05 (ns)	0.19*∗*

Total physical activity score (MET/min/week)^c^	0.09 (ns)	0.27*∗∗∗*

MRS=menopause rating scale, BMI=body mass index, WHR=waist to hip ratio, ns=not significant.

Correlations were Pearson correlation (^c^) or Spearman rank order correlation (^b^) or point biserial correlation (^a^).

Correlations were significant at *∗∗∗∗*<0.001, *∗∗∗*<0.01 and *∗*<0.05.

## Data Availability

The data used to support the findings of this study are available from the corresponding author upon request.
